# The University of California Study of Outcomes in Mothers and Infants (a Population-Based Research Resource): Retrospective Cohort Study

**DOI:** 10.2196/59844

**Published:** 2024-12-03

**Authors:** Rebecca J Baer, Gretchen Bandoli, Laura Jelliffe-Pawlowski, Christina D Chambers

**Affiliations:** 1 Department of Pediatrics University of California San Diego La Jolla, CA United States; 2 Department of Obstetrics, Gynecology, & Reproductive Sciences University of California San Francisco San Francisco, CA United States; 3 UC San Diego Study of Outcomes in Mothers and Infants University of California San Diego La Jolla, CA United States; 4 The California Preterm Birth Initiative University of California San Francisco San Francisco, CA United States; 5 Department of Epidemiology and Biostatistics University of California San Francisco San Francisco, CA United States

**Keywords:** birth certificate, vital statistics, hospital discharge, administrative data, linkage, pregnancy outcome, birth outcome, infant outcome, adverse outcome, preterm birth, birth defects, pregnancy, prenatal, California, policy, disparities, children, data collection

## Abstract

**Background:**

Population-based databases are valuable for perinatal research. The California Department of Health Care Access and Information (HCAI) created a linked birth file covering the years 1991 through 2012. This file includes birth and fetal death certificate records linked to the hospital discharge records of the birthing person and infant. In 2019, the University of California Study of Outcomes in Mothers and Infants received approval to create similar linked birth files for births from 2011 onward, with 2 years of overlapping birth files to allow for linkage comparison.

**Objective:**

This paper aims to describe the University of California Study of Outcomes in Mothers and Infants linkage methodology, examine the linkage quality, and discuss the benefits and limitations of the approach.

**Methods:**

Live birth and fetal death certificates were linked to hospital discharge records for California infants between 2005 and 2020. The linkage algorithm includes variables such as birth hospital and date of birth, and linked record selection is made based on a “link score.” The complete file includes California Vital Statistics and HCAI hospital discharge records for the birthing person (1 y before delivery and 1 y after delivery) and infant (1 y after delivery). Linkage quality was assessed through a comparison of linked files and California Vital Statistics only. Comparisons were made to previous linked birth files created by the HCAI for 2011 and 2012.

**Results:**

Of the 8,040,000 live births, 7,427,738 (92.38%) California Vital Statistics live birth records were linked to HCAI records for birthing people, 7,680,597 (95.53%) birth records were linked to HCAI records for the infant, and 7,285,346 (90.61%) California Vital Statistics birth records were linked to HCAI records for both the birthing person and the infant. The linkage rates were 92.44% (976,526/1,056,358) for Asian and 86.27% (28,601/33,151) for Hawaiian or Pacific Islander birthing people. Of the 44,212 fetal deaths, 33,355 (75.44%) had HCAI records linked to the birthing person. When assessing variables in both California Vital Statistics and hospital records, the percentage was greatest when using both sources: the rates of gestational diabetes were 4.52% (329,128/7,285,345) in the California Vital Statistics records, 8.2% (597,534/7,285,345) in the HCAI records, and 9.34% (680,757/7,285,345) when using both data sources.

**Conclusions:**

We demonstrate that the linkage strategy used for this data platform is similar in linkage rate and linkage quality to the previous linked birth files created by the HCAI. The linkage provides higher rates of crucial variables, such as diabetes, compared to birth certificate records alone, although selection bias from the linkage must be considered. This platform has been used independently to examine health outcomes, has been linked to environmental datasets and residential data, and has been used to obtain and examine maternal serum and newborn blood spots.

## Introduction

### Background

The use of population-based studies for public health surveillance and research has proved highly valuable for perinatal and pediatric epidemiology [1]. These studies frequently use data from birth certificate records, which provide a census of all births occurring within a specific region. Linking birth certificate records to external databases (eg, hospital discharge, environmental pollutant, and census data) can improve the value of population-based studies because it allows researchers to study a wider set of exposures and outcomes that are not available in the vital statistics files. Linkage with hospital records also enhances the measurement of variables that have been underreported on birth certificates, such as comorbidities for the birthing person [2,3], and allows for postnatal follow-up of the mother-child dyad.

With the purpose of studying pregnancy and birth-related outcomes, the California Office of Statewide Health Planning and Development (OSHPD), now known as the California Department of Health Care Access and Information (HCAI), contracted for the creation of linked birth files covering the years 1991 through 2012. The contractor conducted linkages between birth and fetal death certificate records with the hospital discharge records of the birthing person (9 mo before delivery and 1 y after delivery) and infant (1 y after delivery) [4]. These OSHPD datasets were used for many important studies [5-8]. However, file creation was discontinued after 2012, and updated files were needed to examine current data.

In 2015, the University of California Study of Outcomes in Mothers and Infants (SOMI) was created by CDC and GB at the University of California San Diego (UCSD) and LJ-P at the University of California San Francisco (UCSF) [9]. SOMI was developed in collaboration with community and public health partners to predict and promote healthy outcomes in birthing people, infants, and children across California, with a special focus on San Diego County. San Diego County is the second most populous county in California with a diverse racial, ethnic, and economic makeup, including a large population of migrants and refugees. SOMI’s goal is to develop a better understanding of the complex combination of factors—from biological to social and from economic to environmental—that contribute to birthing and child health outcomes. To achieve this goal, the SOMI data platform was created as a foundation of the research in collaboration with the UCSF California Preterm Birth Initiative [10] and the Healthy Outcomes of Pregnancy for Everyone study [11].

In 2019, SOMI received approval from the Committee for the Protection of Human Subjects within California’s Health and Human Services Agency to create updated linked birth files after this work was discontinued by the OSHPD. Subsequently, SOMI gained approval to link additional state databases to enhance the data platform’s utility. Future projects include using the data platform to obtain additional California state data that will provide information about outpatient visits, medication use, and childhood outcomes beyond the first year of life. The SOMI data platform allows multidisciplinary researchers, policy makers, and other stakeholders to visualize and quantify maternal and child health outcomes and related disparities across San Diego County and California and to examine trends over time.

### Objectives

In this paper, we provide an overview of SOMI. The mission of SOMI is to predict and promote healthy outcomes in birthing people, infants, and children in collaboration with community and public health partners. The SOMI platform was created to achieve this goal. This platform connects birth, death, and other health records; biobank specimens; and environmental exposures at the individual or regional level. These data may be used to understand disease etiology, inform current diagnoses and clinical care protocols for infants affected by a variety of birth defects and medical conditions, and inform prevention policy. The SOMI data platform also allows multidisciplinary researchers, policy makers, and other stakeholders to visualize and quantify disparities in child health outcomes across San Diego County and beyond. SOMI scholars have been recruited to use the data platform to advance clinical knowledge and provide important big data experience for emerging researchers.

## Methods

### Overview

Before 2019 (when SOMI received institutional review board approval to create updated linked birth files), projects used OSHPD-created linked birth files as the data source. In 2019, the SOMI data platform was created using the methodology described in the next subsections. These same methods have also been used for the creation of other similar datasets by partners at the UCSF California Preterm Birth Initiative [10,11].

### Data Sources

The primary source data file for the SOMI data platform is the birth cohort file maintained by California Vital Statistics. This file contains live birth certificates, linked infant death certificates within 1 year of birth when a death occurred, and fetal death certificates [12]. Births occurring in California and resulting in a live birth must be registered within 10 days of the birth, regardless of whether it occurred in a hospital, home, or elsewhere. Fetal death certificates are issued for fetal deaths, defined as those where “the fetus does not breathe or show any other evidence of life such as the beating of the heart, pulsation of the umbilical cord, or definite movement of voluntary muscles,” occurring after 20 weeks of gestation [13,14]. Fetal deaths exclude induced abortions and should be registered within 8 days of delivery. Birth certificate records include all California live-born infants and contain demographic information as well as information about pregnancy, labor and delivery, and infant outcomes. Infant death, defined as death within 1 year of birth, is recorded on death certificates, which include information about the cause of death. Between 2005 and 2020, 420,000 to 570,000 live-born infants and 2500 fetal deaths (deaths occurring after 20 weeks of gestation) were recorded annually in California [15].

**Figure 1:**
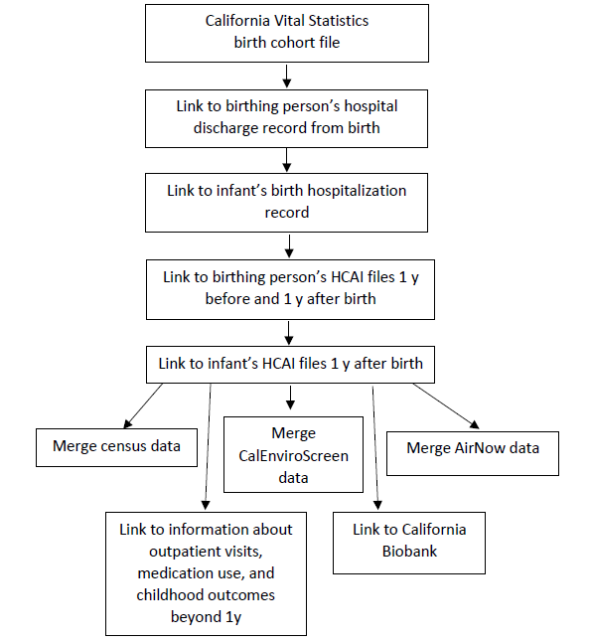
Sample selection: California births from 2011 to 2020 used as the foundation of the University of California Study of Outcomes in Mothers and Infants data platform. HCAI: California Department of Health Care Access and Information.

The second source of data for the SOMI dataset comes from the HCAI, which collects patient discharge, emergency department, and ambulatory surgery center data HCAI [16]. The linked birth file is created through a probabilistic linkage algorithm, developed by the UCSD- and UCSF-based SOMI team and is similar to other published algorithms (Figure 1) [17]. Details are provided in the following subsections.

### Principal File Linkage

#### Link California Vital Statistics Record to Birthing Person’s Hospital Discharge Record for Birth Admission

We identified variables that were available both in California Vital Statistics records and HCAI files. These included the birthing person’s date of birth, hospital where the birth occurred, infant’s date of birth, and sociodemographic and clinical characteristics associated with the birth (Table 1). We used a blocking strategy that involves 2 stages. The first stage identifies possible links for each record based on linking the birthing person’s date of birth and hospital of birth. The second stage scores the possible links within each block (refer to the scoring system presented in Table 1). Scores for each variable were assigned based on the strength of the variable (eg, linked date of birth had a higher score than linked infant sex). Accurate linkages were prioritized over high linkage rates; therefore, a pessimistic linkage method was used to increase the likelihood of true matches. Before performing the linkage, it was determined that a successful link required that (1) the dates of birth for both the birthing person (10 points) and the infant (10 points) must be linked, the hospital (10 points) must be linked, and at least 1 other variable must be linked; or (2) if the dates of birth or hospital did not link exactly, the month and day could be reversed, or the year could differ for the birthing person. After scoring, approximately a subset of matched records with the lowest link score were randomly reviewed by RB to determine whether this requirement resulted in links that seemed accurate based on in-person record review. All match variables were compared among these subsets. On the basis of the point values of these links and a review of individual records, it was determined that a link score of at least 40 was required, in addition to the required matching variables mentioned previously. Although records with a link score of <40 had matching strong variables such as birthing person’s date of birth, infant’s date of birth, and hospital of birth, without further matching information (eg, mode of delivery, race and ethnicity, and county of residence), we were not confident enough to proceed with the match, and these records were discarded. For singleton births, we selected the record with the highest link score (above the cutoff score required) as the final linked record and removed all other records. For multiple births, we linked multiple records to the same birthing person and required that the births be classified as multiple (eg, twin or triplet) births in both the California Vital Statistics and HCAI records. In the rare instance where ≥2 records had the highest link score, the linked record was randomly selected form all records with this link score.

**Table 1:** Scoring system for variables used in the linkage between California Vital Statistics and California Department of Health Care Access and Information (HCAI) files.

Base file: California Vital Statistics	Linked file: HCAI				Link score^a^
	Overall	*ICD-9*^b^ codes	*ICD-10*^c^ codes		
Birthing person’s birth date	Date of birth	—^d^	—	10; 5 if month and day are reversed	
Hospital of birth	Hospital of admission or ED^e^ visit or AS^f^	—	—	10	
Infant’s birth date	Date of birth; age at admission	—	—	10; 5 if month and day are reversed	
Residential zip code	Residential zip code	—	—	5	
Gestational age	—	<29 wk: 765.21, 765.22, 765.23, and 765.24; 29 to <37 wk: 765.25, 765.26, 765.27, and 765.28; 37 to 42 wk: 765.29	<29 wk: P07.2; 29 to <37 wk: O60.1, O60.3, and P07.3; >42 wk: P08.22 and O48	10 if <37 wk, additional 10 if gestation <29 wk, 2 if 37 to 42 wk, and 10 if >42 wk	
Infant sex	Sex	—	—	2	
Payer	Payer	—	—	5	
Race	Race	—	—	5 if any link, additional 5 if American Indian or Alaska Native, Asian or Hawaiian or Pacific Islander, Black, or other race link, and additional 2 if White link	
Ethnicity	Ethnicity	—	—	2	
Residential county	Residential county	—	—	2	
Infant death	Discharge status=died	—	—	10	
Cesarean delivery	—	Procedure code: 74; diagnostic codes: 669.7 and 763.4	Procedure code: 10D; diagnostic codes: O82, P03.4, Z38.01, Z38.31, and Z38.62	5	
Previous cesarean delivery	—	654.2	O34.2	10	
Birthweight	—	<2500 g: 765.01, 765.02, 765.03, 765.04, 765.05, 765.06, 765.07, 765.08, 765.11, 765.12, 765.13, 765.14, 765.15, 765.16, 765.17, and 765.18; ≥2500 g: 765.09 and 765.19	<2500 g: P05.01, P05.02, P05.03, P05.04 P05.05, P05.07 P05.08, P05.11, P05.12, P05.13, P05.14 P05.15, P05.17 P05.18, P07.0, and P07.1; ≥2500 g to <4500 g: P05.09 and P05.19; ≥4500 g: P08.0	10 if birthweight <2500 g, 2 if birthweight is ≥2500 to <4500 g, and 10 if birthweight is ≥4500 g	
Small or large for gestational age	—	656.5, 764.0, 764.1, and 764.9	P05 and O36.5	7	
Hypertension during pregnancy (including preeclampsia and eclampsia)	—	642	O1	10	
Diabetes (preexisting and gestational)	—	648.0, 648.8, and 250	O24	10	
Singleton or twin or multiple gestation	—	Singleton: V27.0; twin: 651.0; multiple gestation: 651.1 and 651.2	Singleton: Z38.0, Z38.1, and Z38.2; twin: O30.0, Z38.3, Z38.4, and Z38.5; multiple gestation: O30.1, O30.2, O38.8, O38.9, Z38.6, Z38.7, and Z38.8	2 for singleton and 10 for twin or other multiple gestation	

^a^The link score is the number of points assigned to individual linkage if there is concordance between the variable values in California Vital Statistics and HCAI files. The possible range of link scores is from 0 to 148.

^b^*ICD-9*: International Classification of Diseases, Ninth Revision.

^c^*ICD-10*: International Classification of Diseases, Tenth Revision.

^d^Not applicable.

^e^ED: emergency department.

^f^AS: ambulatory surgery.

As we allowed for the month and day to be reversed or for the year to differ in the birthing person’s date of birth, we continued linkage using multiple passes and different linking variables. We repeated the process for any unlinked records after the first pass using different variables in the blocking step of the linkage (pass B: infant’s date of birth and patient zip code, pass C: infant’s date of birth and hospital of birth, pass C: birthing person’s date of birth and patient zip code, pass E: patient zip code and hospital of birth, and pass F: preterm birth and infant’s date of birth). Before each pass, previously linked records were removed. Records without a link after pass F remained unlinked.

#### Link Birth Certificate to Infant’s Hospital Discharge Record for Birth Admission

Hospital discharge records were restricted to records with an indication of pregnancy or birth (major diagnostic category code 14; *International Classification of Diseases* [*ICD*; *ICD-9*] codes V23 or any 640-679; *ICD-10* O1, O2, O3, O4, O6, O7, O8, and O9). We used the same method to link the birth certificate records to the infant’s hospital discharge record for the birth admission, with different variables for the first blocking step (pass A: hospital of birth and infant’s date of birth; passes B, C, and D repeated pass A to obtain duplicates for twins or multiples; pass E: infant’s date of birth and patient zip code; pass F: repeat pass E to obtain duplicates for twins or multiples; pass G: patient zip code and infant sex; pass H: patient zip code and payer category; and pass I: patient zip code and hospital of birth). Records without linking infant sex were not considered links regardless of link score. After scoring, a subset of records were randomly examined by RB, and a cutoff score of 31 was determined to balance sensitivity and specificity to ensure accurate linkage (of note, this was only linking infant to infant because the birthing person’s date of birth was not available for infant hospital discharge records, hence the lower match score threshold). As fewer variables were available for comparison between the 2 infant datasets, the link score was set lower than for the birthing person. We note that if twins or multiples shared the same sex, birthweight range, and mode of delivery, and all survived, it was impossible to decipher which record should be linked, and the records were randomly assigned.

#### Linkage to Additional Hospital Discharge, Emergency Department, and Ambulatory Surgery Records for Birthing Persons and Infants

This step enables multiple linkages over time because a person might be admitted multiple times. It allows an evaluation of hospital visits during pregnancy and readmissions before or after delivery. The methodology for this was similar to the prior linkages; however, now encrypted social security numbers (SSNs) available within the HCAI files were used in addition to the variables listed in Table 1 to strengthen the link. Encrypted SSNs are present for approximately 80% of people giving birth and <10% of infants. As an SSN can potentially be used by different individuals, matching SSNs alone was not considered a match. Other matching variables, in addition to matching SSNs, were required. Moreover, the algorithm considers variables in previous HCAI records (from birth admission) to strengthen the link.

### Evaluation of Linkage Quality

#### Performance of Linkage

We evaluated the performance of the linkage by estimating the percentage of birth and fetal death certificate records that were successfully linked to (1) hospital records for the birthing person, (2) hospital records for the infant, and (3) hospital records for both individuals. We estimated these percentages for the full cohort, as well as by year, sociodemographic characteristics, and pregnancy outcomes.

#### Comparison With Previous Linkage Methodology

Next, we compared our dataset to the one created using the prior OSHPD methodology. Prior files were created using probabilistic linkage [18,19], although the code was not available for comparison. For the evaluation of linked files, we selected the 2 most recent years of the OSHPD linked birth files: 2011 and 2012. By merging on the California Vital Statistics birth state file number (SFN), we compared the individual records that were linked to determine whether we were linking to the same patient. We compared each variable in the record between the 2 files, and *exact* links were considered the same patient. We also compared the sociodemographic and clinical characteristics of those in the linked and unlinked populations because unequally distributed unlinked records could result in bias [20]. Finally, we reviewed records linked by the previously used OSHPD methodology and the SOMI methodology to determine whether our algorithm needed modification to improve the number and percentage of true matches.

### Evaluation of Data Quality and Bias

To evaluate the potential for sampling bias due to the exclusion of unlinked records, we analyzed the prevalence of preterm birth among the live-born infant cohort and separately by race and ethnicity. We first evaluated the prevalence of preterm birth using only California Vital Statistics data and then in the cohort of linked records. We repeated this with stratification by race and ethnicity. Preterm live birth was defined as gestation of 20 to <37 completed weeks. Gestational age at birth and race and ethnicity data were obtained from birth certificate records.

To evaluate the gain in information from querying hospital discharge records in addition to California Vital Statistics, we estimated the prevalence of several characteristics in the linked cohort first from only the information provided on the birth record and then separately from the presence of the characteristics on the hospital discharge record or the birth record.

### Analytic Software

All linkages and analyses were performed using SAS (version 9.4; SAS Institute Inc).

### Data Available in the Principal Data Platform

From the California Vital Statistics records, the SOMI platform contains information from the vital statistics records for the birthing person and nonbirthing person, typically the father (race and ethnicity, education, and occupation); residence (address, zip code, and census tract); payer for prenatal care and expected payer for delivery; prepregnancy and delivery weight; height; number of prenatal care visits; the month prenatal care began; the mode of delivery; gestational age at delivery; singleton or twin or multiple; infant’s birth weight; infant’s date of birth; complications during pregnancy and labor and delivery; and abnormal conditions of the newborn. Birth certificate files contain a birth SFN (unique by year). Linked infant deaths have an *ICD* code for the cause of death and a death SFN (unique by year) [12].

The HCAI files (patient discharge, emergency department, and ambulatory surgery center data) provide date of admission and discharge, *ICD* diagnoses and procedure codes, principal language spoken, and discharge status [21].

### Build Out of the Data Platform

**Figure 2:**
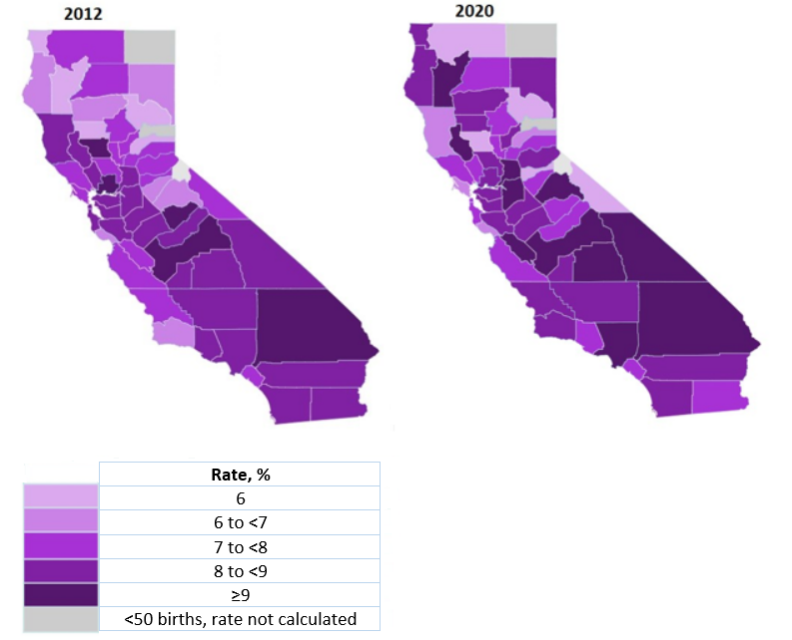
Preterm birth (<37 wk gestation) rates by California county in 2012 and 2020.

To improve the SOMI platform’s utility, additional datasets have been merged on residential zip codes or census tracts. These datasets include CalEnviroScreen [22], US census data [23], and AirNow [24]. California birth certificates include a text field for occupation and business or industry for the person giving birth (typically the mother) and the person not giving birth (typically the father). As these fields include different ways to describe occupation (eg, teacher or elementary school teacher) and may include typos, misspellings, and abbreviations, the SOMI data platform has cleaned these variables using the Occupational Safety and Health Administration algorithm, allowing for analysis of birth outcomes by occupation (eg, firefighter, bartender, or dry cleaner). In addition, the platform has the potential to merge indicators of redlining, food deserts, traffic, walkability, and any other data sources with geographic features (eg, zip code, address, and geocodes; Figure 2).

### Biological Specimens

With the birth SFN provided in the California Vital Statistics file, SOMI data platform is able to identify individuals of interest to be able to obtain biospecimens from the California Biobank (biospecimens in the biobank have birth SFN linked) [25,26]; for example, the authors of this study were awarded a genomic project, funded by the National Institutes of Health, examining unexplained causes of infant deaths in San Diego County. Through the SOMI platform, all infants who died in the first year of life in San Diego County over a 10-year period were identified, and newborn blood spots stored in the California Biobank were requested. Whole-genome sequencing was performed and is now being used in combination with SOMI data on environmental, demographic, geographic, pregnancy, and infant characteristics to understand the proportion of cases that might be explained by some combination of factors and potentially prevented [25-27].

### Ethical Considerations

Methods and protocols for the study were approved by the Committee for the Protection of Human Subjects within California’s Health and Human Services Agency (project 2019-025) and the institutional review board at the UCSD (project 190892). This protocol was first approved in 2019 and has received annual continuing approval to date. The protocol includes a waiver of informed consent. All identifying data are stored behind secure UCSD firewalls in accordance with the requirements of the Committee for the Protection of Human Subjects, California Vital Statistics, and the HCAI. As the data used in this study come from an administrative database, no compensation was provided to individuals whose data were included in the files.

## Results

### Principal File Linkage

There were 8,084,212 deliveries, including 8,040,000 (99.45%) live births and 44,212 (0.55%) fetal deaths, in California between 2005 and 2020. Using the SOMI linkage methodology, 92.38% (7,427,738/8,040,000) of the California Vital Statistics live birth records were linked to the HCAI records for the birthing person, 95.53% (7,6880,597/8,040,000) of the California Vital Statistics birth records were linked to the HCAI records for the infant, and 90.61% (7,285,345/8,040,000) of the California Vital Statistics birth records were linked to the HCAI records for both the birthing person and the infant. Of the 44,212 fetal deaths, 33,355 (75.44%) had HCAI records linked to the birthing person. There was some variation in the percentage linked by year (Table 2). The percentage of records linked did not vary by the age of the birthing person but was poor 399/1788, 22.3%) for individuals whose age was unknown. There were some differences by race and ethnicity. Records where the birthing person was Asian or Hispanic had a higher proportion of linkage. Records where the birthing person was American Indian or Alaska Native, Hawaiian or Pacific Islander, or did not have a race reported had a lower rate of linkage. Approximately 73.31% (31,356/42,770) of the preterm infants born at <28 weeks had records linked to both the infant and the birthing person, whereas 91.07% (6,529,658/7,169,856) of the term infants born at 37 to 42 weeks had both linkages (Table 2).

**Table 2:** Percentage of California Vital Statistics records linked to California Department of Health Care Access and Information (HCAI) records by year and sociodemographic characteristics.

Characteristics^a^		Population size^b^: total live births, n (%)	California Vital Statistics records linked to HCAI records for birthing person, n (%)	California Vital Statistics records linked to HCAI records for infant, n (%)	California Vital Statistics records linked to HCAI records for birthing person and infant, n (%)
Sample		8,040,000 (100)	7,427,738 (92.38)	7,680,597 (95.53)	7,285,345 (90.61)
**Year of birth^c^**					
	2005	548,636 (100)	510,628 (93.07)	524,373 (95.58)	498,873 (90.93)
	2006	562,467 (100)	526,119 (93.54)	537,769 (95.52)	513,384 (91.27)
	2007	566,333 (100)	532,424 (94.01)	543,769 (96.02)	521,918 (92.16)
	2008	551,871 (100)	523,929 (94.94)	530,511 (96.13)	514,140 (93.16)
	2009	527,044 (100)	500,695 (95.00)	506,791 (96.16)	492,003 (93.35)
	2010	509,630 (100)	481,128 (94.41)	486,940 (95.55)	472,147 (92.65)
	2011	501,816 (100)	475,649 (94.79)	480,747 (95.80)	466,869 (93.04)
	2012	502,299 (100)	477,415 (95.05)	480,655 (95.69)	469,026 (93.38)
	2013	493,596 (100)	467,390 (94.69)	468,902 (95)	465,539 (92.49)
	2014	501,600 (100)	472,480 (94.19)	478,560 (95.41)	463,828 (92.47)
	2015	490,675 (100)	456,548 (93.04)	467,629 (95.30)	448,169 (91.34)
	2016	487,869 (100)	446,967 (91.62)	467,532 (95.83)	440,588 (90.31)
	2017	470,888 (100)	427,837 (90.86)	448,016 (95.14)	420,161 (89.23)
	2018	456,244 (100)	392,392 (86)	435,054 (95.36)	386,197 (84.65)
	2019	447,869 (100)	380,522 (84.96)	425,256 (94.95)	373,159 (83.32)
	2020	421,163 (100)	355,615 (84.44)	398,598 (94.64)	348,344 (82.71)
**Age (y) of birthing person^c^**					
	<18	168,513 (100)	158,858 (94.27)	163,249 (96.88)	155,431 (92.24)
	18-34	6,261,136 (100)	5,782,834 (92.36)	5,973,141 (95.40)	5,671,962 (90.59)
	>34	1,608,563 (100)	1,485,647 (92.36)	1,543,219 (95.94)	1,457,591 (90.61)
	Missing	1788 (100)	399 (22.3)	988 (55.3)	361 (20.2)
**Payer for delivery^c^**					
	Private	3,828,332 (100)	3,561,673 (93.03)	3,711,571 (96.95)	3,497,160 (91.35)
	Public	3,599,004 (100)	3,415,751 (94.91)	3,507,027 (97.44)	3,353,669 (93.18)
	Self-pay	243,859 (100)	195,436 (80.14)	199,967 (82)	189,570 (77.74)
	Other^d^	354,328 (100)	243,461 (68.71)	249,756 (70.49)	234,071 (66.06)
	Unknown or missing	14,477 (100)	11,417 (78.86)	12,276 (84.80)	10,875 (75.12)
**Race and ethnicity^c^**					
	American Indian or Alaska Native	27,129 (00)	24,187 (89.16)	25,283 (93.20)	23,672 (87.26)
	Asian	1,056,358 (100)	992,222 (93.93)	1,022,442 (96.79)	976,526 (92.44)
	Black	411,552 (100)	372,940 (90.62)	388,477 (94.39)	365,143 (88.72)
	Hawaiian or Pacific Islander	33,151 (100)	4,627 (89.16)	31,028 (93.60)	28,601 (86.27)
	Hispanic	3,910,385 (100)	3,669,072 (93.83)	3,785,908 (96.82)	3,603,171 (92.14)
	White, not Hispanic	2,240,243 (100)	2,030,737 (90.65)	2,095,086 (93.52)	1,987,431 (88.71)
	Other	5155 (100)	4,627 (89.76)	4,923 (95.50)	4,520 (87.68)
	≥2	167,040 (00)	148,692 (89.02)	156,263 (93.55)	145,657 (87.20)
	Unknown	188,987 (100)	156,024 (82.56)	171,187 (90.58)	150,624 (79.70)
**Education level (y)^c^**					
	<12	1,603,261 (100)	1,532,999 (95.62)	1,561,759 (97.41)	1,503,884 (93.80)
	12	1,960,415 (100)	1,808,044 (92.23)	1,869,660 (95.37)	1,773,120 (90.45)
	>12	4,122,006 (100)	3,778,745 (91.67)	9,919,057 (95.17)	3,709,154 (89.98)
	Missing	354,318 (100)	307,950 (86.91)	330,121 (91.17)	299,187 (84.44)
**Gestational age (wk)^c^**					
	20-27	42,770 (100)	36,825 (86.10)	35,779 (83.65)	31,356 (73.31)
	28-31	65,147 (100)	56,588 (86.86)	55,726 (85.54)	48,944 (75.13)
	32-36	608,456 (100)	522,747 (90.84)	582,235 (95.69)	538,043 (88.43)
	37-42	7,169,856 (100)	6,641,573 (92.63)	6,862,280 (95.71)	6,529,658 (91.07)
	43-44	91,061 (100)	84,070 (92.32)	85,886 (94.32)	82,506 (90.61)
	Missing	62,710 (100)	55,935 (89.20)	58,691 (93.59)	54,848 (87.45)
**Gestation^c^**					
	Singleton	7,787,531 (100)	7,222,886 (92.75)	7,439,451 (95.53)	7,085,257 (90.98)
	Twins	243,585 (100)	198,052 (81.31)	233,023 (95.66)	193,574 (79.47)
	Other multiples	8708 (100)	6,800 (78.09)	8,115 (93.19)	6,514 (74.80)
	Missing	176 (100)	0 (0)	<11 (4.55)	0 (0)
Infant death^c^		37,975 (100)	32,760 (86.27)	33,851 (89.14)	30,297 (79.78)
Fetal death		44,212 (100)	33,355 (75.44)	—^e^	—

^a^Population size determined by California Vital Statistics birth and fetal death certificates.

^b^All characteristics variables (including infant death) obtained from California Vital Statistics records only.

^c^Restricted to live births with a state file number, deduplicated by year and state file number.

^d^Includes medically unattended birth, TRICARE or Civilian Health and Medical Program of the Uniformed Services, other government programs, other payer, and Indian Health Service.

^e^Linkage to infant not possible.

### Performance of Linkage

The linked population was more similar to the total population than the unlinked population, as one would expect given the high linkage rate. The population without a link to a birthing person had a smaller percentage of people using Medi-Cal (California’s Medicaid program), individuals of Hispanic ethnicity, and singleton births compared to the population linked to the records of the birthing person, as well as a larger percentage of birthing people delivering at 20 to <37 weeks of gestation and pregnant with twins. When examining the differences in the births linked to an infant hospital record, the findings were similar (Table 3).

**Table 3:** Characteristics of records in California Vital Statistics with stratification based on linkage to California Department of Health Care Access and Information recordsa.

California Vital Statistics variables				Population size: total live births (n=8,040,000), n (%)		Birthing person					Infant		
						Linked (n=7,427,738), n (%)		Unlinked (n=618,938), n (%)		Linked (n=7,680,597), n (%)			Unlinked (n=366,079), n (%)
**Sociodemographics**													
	**Age (y) of birthing person**												
		<18	168,513 (2.1)		158,858 (2.14)		9655 (1.58)		163,249 (2.13)			5264 (1.46)	
		18-34	6,261,136 (77.87)		5,782,834 (77.85)		478,302 (78.12)		5,973,141 (77.77)			287,995 (80.13)	
		>34	1,608,563 (20.01)		1,485,647 (20)		122,916 (20.08)		1,543,219 (20.09)			65,344 (18.18)	
		Missing	1788 (0.02)		399 (0.01)		1389 (0.23)		988 (0.01)			800 (0.22)	
	**Payer for delivery**												
		Medi-Cal	3,599,004 (44.76)		3,415,751 (45.99)		183,253 (29.93)		3,507,027 (45.66)			91,977 (25.59)	
		Private	3,828,332 (47.62)		3,561,673 (487.95)		266,659 (43.55)		3,711,571 (48.32)			116,761 (32.49)	
		Self-pay	243,859 (3.03)		195,436 (2.63)		48,423 (7.91)		199,967 (2.60)			43,892 (12.21)	
		Other	354,328 (4.41)		243,461 (3.28)		110,867 (18.11)		249,756 (3.25)			104,572 (29.10)	
		Unknown or missing	14,477 (0.18)		11,417 (0.15)		3060 (0.50)		12,276 (0.16)			2201 (0.61)	
	**Education level (y)**												
		<12	1,603,261 (19.94)		1,532,999 (20.64)		70,262 (11.48)		1,561,759 (20.33)			41,502 (11.55)	
		12	1,960,415 (24.38)		1,808,044 (24.34)		152,371 (24.89)		1,869,660 (24.33)			90,755 (25.25)	
		>12	4,122,006 (51.27)		3,778,745 (50.87)		343,261 (56.06)		3,919,057 (51.03)			202,949 (56.47)	
		Missing	354,318 (4.41)		307,950 (4.15)		46,368 (7.57)		330,121 (4.30)			24,197 (6.73)	
	**Race and ethnicity**												
		American Indian or Alaska Native	27,129 (0.34)		24,187 (0.33)		2942 (0.48)		25,283 (0.33)			1846 (0.51)	
		Asian	1,056,358 (13.14)		992,222 (13.36)		64,136 (10.48)		1,022,442 (13.31)			33,916 (9.44)	
		Black	411,552 (5.12)		372,940 (5.02)		38,612 (6.31)		388,477 (5.06)			23,075 (6.42)	
		Hawaiian or Pacific Islander	33,151 (0.41)		29,237 (0.39)		3914 (0.64)		31,028 (0.40)			2123 (0.59)	
		Hispanic	3,910,385 (48.64)		3,669,072 (49.40)		241,313 (39.41)		3,785,908 (49.29)			124,477 (34.63)	
		White, not Hispanic	2,240,243 (27.86)		2,030,737 (27.34)		209,506 (34.22)		2,095,086 (27.28)			145,157 (40.39)	
		Other	5155 (0.06)		4627 (0.06)		528 (0.09)		4923 (0.06)			232 (0.06)	
		≥2	167,040 (2.08)		148,692 (2)		18,348 (3)		156,263 (2.03)			10,777 (3)	
		Unknown	188,987 (2.35)		156,024 (2.10)		32,963 (5.38)		171,187 (2.23)			17,800 (4.95)	
**Pregnancy and birth outcomes**													
	**Gestational age (wk)**												
		20-27	42,770 (0.53)		36,825 (0.50)		5,945 (0.97)		35,779 (0.47)			6,991 (1.95)	
		28-31	65,147 (0.81)		56,588 (0.76)		8,559 (1.40)		55,726 (0.73)			9,421 (2.62)	
		32-36	608,456 (7.57)		552,747 (7.44)		55,709 (9.10)		582,235 (7.58)			26,221 (7.30)	
		37-42	7,169,856 (89.18)		6,641,573 (89.42)		528,283 (86.28)		6,862,280 (89354)			307,576 (85.58)	
		43-44	91,061 (1.13)		84,070 (1.13)		6991 (1.14)		85,886 (1.12)			5175 (1.44)	
		Missing	62,710 (0.78)		55,935 (0.75)		6775 (1.11)		58,691 (0.76)			4019 (1.12)	
	**Gestation**												
		Singleton	7,787,531 (96.86)		7,222,886 (97.24)		564,645 (92.22)		7,439,451 (96.86)			348,080 (96.85)	
		Twins	243,585 (3.03)		198,052 (2.67)		45,533 (7.44)		233,023 (3.03)			10,562 (2.94)	
		Other multiples	8708 (0.11)		6800 (0.09)		1908 (0.31)		8115 (0.11)			593 (0.16)	
		Missing	176 (0)		0 (0)		176 (0.03)		<11 (0)			168 (0.05)	
Infant death				37,975 (0.47)		32,760 (0.44)		5215 (0.85)		33,851 (0.44)			4124 (1.15s)

^a^Characteristics variables obtained from California Vital Statistics records.

### Comparison With Previous Linkage Methodology

During 2011 and 2012, California Vital Statistics recorded 1,009,197 births. SOMI methodology linked 92.74% (935,895/1,009,197) of these records to the birthing person and live-born infant compared to 96.04% (969,258/1,009,197) for the OSHPD methodology (determined from the SOMI files obtained from the HCAI). Of the 928,459 records that had a link using both linkage methodologies, 923,705 (99.49%) were linked to the same HCAI record for the birthing person, 907,745 (97.77%) were linked to the same HCAI record for the infant, and 903,183 (97.28%) were linked to the same HCAI record for the birthing person and the infant.

There were 40,773 records with an OSHPD link between the birthing person and an infant who did not have this same link in the SOMI file. Of these 40,773 records, the SOMI file had 23,001 (56.41%) infants with a link between California Vital Statistics and HCAI records but had no link to a birthing person. Similarly, SOMI had 32.33% (13,182/40,773) of the birthing persons having a California Vital Statistics and HCAI link but no link to an infant. In 4590 (11.26%) of these 40,773 records, SOMI had no link for birthing person or infant between California Vital Statistics and the HCAI and also had no link between the birthing person and infant.

### Data Source of Ascertainment: HCAI Versus California Vital Statistics

The records from HCAI data had greater capture of all variables compared to California Vital Statistics, with the exception of infant death, where both sources had similar capture rates. When only considering periviable births of between 22 and 23 weeks of gestation, the HCAI percentage of infant deaths was smaller than that reported in California Vital Statistics. Querying both data sources resulted in percentages consistent with those expected in the general population (Table 4).

**Table 4:** Variable ascertainment by source (California Vital Statistics or California Department of Health Care Access and Information [HCAI]) among all linked records.

			Variable ascertainment source				
			California Vital Statistics only, n (%)		HCAI only, n (%)		California Vital Statistics or HCAI, n (%)
Gestational diabetes (n=7,285,345)			329,128 (4.52)		597,534 (8.2)		680,757 (9.34)
Preexisting diabetes (n=7,285,345)			50,279 (0.69)		100,794 (1.38)		130,097 (1.79)
Prepregnancy hypertension (n=7,285,345)			47,399 (0.65)		102,893 (1.41)		125,319 (1.72)
Gestational hypertension (pregnancy-induced hypertension and preeclampsia; n=7,285,345)			206,736 (2.84)		463,834 (6.37)		516,346 (7.09)
Eclampsia (n=7,285,345)			5697 (0.08)		6498 (0.09)		11,584 (0.16)
Placental abruption (n=7,285,345)			25,509 (0.35)		70,672 (0.97)		79,420 (1.09)
Chorioamnionitis (n=7,285,345)			38,969 (0.53)		183,260 (2.52)		189,873 (2.61)
**Birth defects (n=7,285,345)**							
	Neural tube defect	772 (0.01)		3333 (0.95)		3626 (0.05)	
	Gastroschisis or omphalocele	1312 (0.02)		6,137 (0.08)		6458 (0.09)	
	Cleft lip or palate	3056 (0.04)		13,913 (0.19)		15,733 (0.22)	
	Major heart defect	1126 (0.02)		27,822 (0.38)		28,410 (0.39)	
Among live births (n=7,285,345), infant death			30,297 (0.42)		30,964 (0.43)		36,440 (0.5)
Among infants born between 22 and 23 wk (n=5724), infant death			4355 (76.08)		4263 (74.48)		4380 (76.52)

### Use Case: Preterm Live Birth Prevalence in Linked Versus Unlinked Files

The preterm live birth rate in the California Vital Statistics records was 8.91% (716,331/8,040,000), and it was 8.49% (285,345/618,325) in the linked cohort (Table 5). The rate was most altered for records where the birthing person selected “not stated/unknown” for their race (21,582/188,987, 11.42% in the California Vital Statistics file vs 15,455/150,624, 10.26% in the linked file) and least altered for people who selected “White, non-Hispanic” (186,166/2,240,243, 8.31% in the California Vital Statistics file vs 159,191/1,987,431, 8.01% in the linked file).

**Table 5:** Prevalence of preterm live births for the population in the California Vital Statistics records and linked dataset by race and ethnicity.

Race and ethnicity	California Vital Statistics only		Linked dataset	
	Population, n	Preterm live births (20-37 wk gestation), n (%)	Population, n	Preterm live births (20-37 wk gestation), n (% preterm births)
Sample	8,040,000	716,331 (8.91)	7,285,345	618,325 (8.49)
American Indian or Alaska Native	27,129	2818 (10.39)	23,672	2350 (9.93)
Asian	1,056,358	89,311 (8.45)	976,526	79,631 (8.15)
Black	411,552	52,459 (12.75)	365,143	44,936 (12.31)
Hawaiian or Pacific Islander	33,151	3354 (10.12)	28,601	2724 (9.52)
Hispanic	3,910,385	344,322 (8.81)	3,603,171	300,438 (8.34)
White, not Hispanic	2,240,243	186,166 (8.31)	1,987,431	159,191 (8.01)
Other	5155	555 (10.77)	4520	448 (9.91)
≥2	167,040	15,764 (9.44)	145,657	13,152 (9.03)
Unknown	188,987	21,582 (11.42)	150,624	15,455 (10.26)

## Discussion

### Principal Findings

#### Overview

In this paper, we described the creation of the SOMI data platform, an administrative cohort derived from California birth and fetal death records. Although our linkage is not identical to the previous linked files created by the OSHPD, we demonstrate that the linked records are similar, as seen by the 97.28% (903,183 /928,459) concurrence between both infant and birthing person. We have demonstrated quality linkages for 90.61% (7,285,345/8,040,000) of the population. We did not see evidence of selection bias for a number of measured variables, including infant’s year of birth, gestational age at birth, and birthing person’s education level.

#### Interpretations, Implications, and Comparisons to Existing Literature

Leveraging birth certificate data to answer research questions has several advantages. First, it makes use of a population-based cohort of existing data, which may be necessary when primary data collection is unrealistic or too costly [20,28]. Second, capturing the entire population improves the generalizability of the research findings. Finally, large sample sizes permit the study of rare health outcomes (eg, periviable birth and birth defects) [28]. Birth certificate files have limitations when used in isolation. It is widely accepted that recorded information, such as birth defects, preeclampsia, and premature rupture of the membranes, is profoundly underascertained [2,3,29,30]. By linking these data to external data sources, variable ascertainment can be improved, and additional factors can be measured; for example, linkage between birth records and hospital discharge records facilitates the measurement of additional health factors such as maternal substance use disorder, mental health illness, infant resuscitation, and complications of prematurity (eg, intraventricular hemorrhage and bronchopulmonary dysplasia). However, linkage creation may lead to selection bias if the linkage is differential across groups.

There are many different methods that can be used to create these linkages. Some are deterministic, requiring perfect links across key variables to link records; others are probabilistic, linking records based on the probability that the records belong to the same individual. Researchers must decide which variables to include in the linkage, what characteristics to prioritize, and how stringent to be in determining a link. These subjective decisions determine the proportion of people who end up in the linked database and who, ultimately, are included in research studies. Thus, linkage procedure can affect both the external validity (who is represented in the data and who is not) and the internal validity (measurement error, unmeasured confounding variables, and selection bias) of the study [20,31]. Our linkage rate is slightly lower than that of the previous OSHPD files (935,895/1,009,197, 92.74% vs 969,258/1,009,197, 96.04%). While the starting populations differed somewhat—the OSHPD files excluded fetal deaths and multiple births [18], whereas the SOMI files included these records—there were differences in the linkage rates observed that were not explained by differences in the starting samples. Importantly, our record-by-record review of the 4590 individuals who were in the OSHPD matched file but were not in the SOMI matched file suggested that these were likely false matches (given missing or highly discrepant information) [16]. As such, it is possible that some other identifier was available for the linkage of this previous OSHPD set, and, in turn, these records could in fact be true matches. Nevertheless, based on the data available to the SOMI team and our commitment to link quality over quantity, no adaptations to our linkage methodology were made as a result of these comparisons.

As there are not many publications on the creation of datasets such as this one, it is difficult to compare our methodology, linkage rates, and data utility to those of other works. In 2013, Salemi et al [32] published on a similar dataset created for births in Florida. Their linkage rate for the 2.3 million birth certificate records to the infant birth hospitalization record was 92.1%. Similar to our study, they reported possible bias in linkage rates. The US government maintains several databases that can be used for maternal and child health research, such as the Pregnancy Risk Assessment and Monitoring System [33] and the Healthcare Cost and Utilization Project [34], both of which contain national data; however, both databases have limitations, including restricted capacity for merging additional data.

### Key Findings to Date

To date, in partnerships with investigators across the country, SOMI has published >50 manuscripts with important findings on maternal and child health (highlights are presented in Table 6). The large population within the data platform has enabled the examination of rare diagnoses such as alcohol use disorder during pregnancy and infant heart defects, cannabis use disorder and infant death, postpartum suicidal behaviors, and infant readmissions associated with air pollution exposure.

**Table 6:** Key University of California Study of Outcomes in Mothers and Infants (SOMI) published projects as of October 2024.

Study (role of first author)	Title	Key findings
Delker et al [35] (postdoctoral fellow)	Prenatal cannabis use disorder and gastroschisis in California, 2007-19	A positive association between cannabis exposure and gastroschisis
Dhital et al [36] (collaboration)	Maternal cardiovascular events in autoimmune rheumatic diseases and antiphospholipid syndrome pregnancies	Pregnancies affected by autoimmune rheumatic diseases and antiphospholipid syndrome were at increased risk of cardiovascular events
Fall et al [37] (collaboration)	Racial and ethnic inequities in therapeutic hypothermia and neonatal hypoxic-ischemic encephalopathy: a retrospective cohort study	Black infants with neonatal hypoxic-ischemic encephalopathy were significantly less likely to receive therapeutic hypothermia; Black infants also had significantly increased risk of some adverse outcomes
Teyton et al [38] (predoctoral fellow)	Disparities in the impact of heat wave definitions on emergency department visits during the first year of life among preterm and full-term infants in California	Infants were at increased risk of an emergency department visit with exposure to all heat wave definitions
Abe et al [39] (collaboration)	Maternal mental health diagnoses and infant emergency department use, hospitalizations, and death	Maternal mental health diagnoses were associated with infant acute emergency department visits, hospitalization, and death
Wang et al [40] (fellow)	Maternal penicillin allergy and infant outcomes: results from a large, administrative cohort	Maternal penicillin allergy diagnosis was associated with increased risk for prolonged hospitalization and readmission within the first week of life for newborn infants of mothers colonized with group B *Streptococcus*
Schellenberg et al [41] (predoctoral fellow)	Pregnancy characteristics and outcomes among birthing individuals with a diagnosis of fetal alcohol syndrome	There were 35 babies born to 30 individuals with a diagnosis of fetal alcohol syndrome between 2005 and 2021 (0.5 per 100,000 live births); the prevalence of births to individuals with a diagnosis of fetal alcohol syndrome increased over the period. Infants of individuals with fetal alcohol syndrome were more likely to be born prematurely or small for gestational age and be admitted to the neonatal intensive care unit
Baer et al [42] (SOMI staff)	Adverse live-born pregnancy outcomes among pregnant people with anorexia nervosa	Anorexia nervosa diagnosis during pregnancy was associated with anemia, preterm labor, oligohydramnios, severe maternal morbidity, and having a small-for-gestational-age or low-birthweight infant, as well as preterm birth between 32 and 36 weeks with spontaneous preterm labor in comparison with people without an eating disorder diagnosis; gestational weight gain below the ACOG^a^ recommendation mediated 38.89% of the excess in preterm births and 40.44% of the excess in low-birthweight infants
Strouse et al [43] (collaboration)	Racial and ethnic disparities in the risk of preterm birth among women with systemic lupus erythematosus or rheumatoid arthritis	Black, Hispanic, and Asian women with systemic lupus erythematosus were 1.3 to 1.5 times more likely to have a preterm birth compared to non-Hispanic White women; Black women with rheumatoid arthritis were 2.0 to 2.4 times more likely to have a preterm birth compared to Asian, Hispanic, or non-Hispanic White women
Delker et al [44] (postdoctoral fellow)	Adverse perinatal outcomes and postpartum suicidal behavior in California, 2013-2018	The prevalence of postpartum suicidal ideation and attempt increased from 2013 to 2018; people with postpartum suicidal behavior were younger, had less education, and were more likely to live in rural areas; a greater proportion of those with postpartum suicidal behavior were Black and publicly insured; severe maternal morbidity, neonatal intensive care unit admission, and fetal death were associated with greater risk of suicidal ideation and attempt
Ding et al [26] (collaboration)	Scalable, high quality, whole genome sequencing from archived, newborn, dried blood spots	Archived dried blood spots seem to be a suitable sample type for whole-genome sequencing in population genomic studies
Teyton et al [45] (collaboration)	Exposure to air pollution and emergency department visits during the first year of life among preterm and full-term infants	Increasing PM_2.5_^b^ exposure was associated with an increased emergency department visit risk for both preterm and full-term infants during the first year of life
Owen et al [25] (medical student)	Reclassification of the etiology of infant mortality with whole-genome sequencing	The association of genetic diseases with infant mortality was higher than previously recognized; in 5 of 7 infants in whom genetic diseases were identified post mortem, death might have been avoided had rapid, diagnostic whole-genome sequencing been performed at the time of symptom onset or regional intensive care unit admission
Bandoli et al [46] (SOMI staff)	Prenatal cannabis use disorder and infant hospitalization and death in the first year of life	The incidence of infant death in the first year of life was greater among those with a maternal cannabis use disorder diagnosis than among those without; the increased risk estimates were attributable to perinatal conditions and sudden unexpected infant death; after adjustment, there was no increased risk of infant hospitalizations or emergency department visits
Bandoli et al [27] (SOMI staff)	Maternal, infant, and environmental risk factors for sudden unexpected infant deaths: results from a large, administrative cohort	Multiparity, maternal depression, substance-related diagnosis, cannabis-related diagnosis, prenatal nicotine use, preexisting hypertension, preterm delivery, infant with a major malformation, respiratory distress syndrome, and select environmental factors were all associated with sudden unexpected infant death
Bandoli et al [47] (SOMI staff)	Risk factors for neonatal encephalopathy in late preterm and term singleton births in a large California birth cohort	Substance-related diagnosis, preexisting diabetes, preeclampsia, and maternal infection were associated with a 2-fold increase in risk of neonatal encephalopathy; maternal overweight and obesity, nulliparity, advanced maternal age, depression, gestational diabetes or hypertension, and short or long gestations also predicted neonatal encephalopathy; young maternal age, Asian race and Hispanic ethnicity, and cannabis-related diagnosis lowered risk of neonatal encephalopathy
Harvey et al [48] (MD and PhD student)	Association of alcohol use diagnostic codes in pregnancy and offspring conotruncal and endocardial cushion heart defects [48]	Alcohol-related diagnostic codes in pregnancy were associated with an increased risk of an offspring with a congenital heart disease, with a particular risk for endocardial cushion and conotruncal defects
Araneta et al [49] (collaboration)	Health advantages and disparities in preterm birth among immigrants despite disparate sociodemographic, behavioral, and maternal risk factors in San Diego, California [49]	Black migrants had lower preterm birth prevalence compared to US-born Blacks, but this immigrant advantage was not observed in other racial and ethnic groups; compared to US-born White individuals, Somali migrants had significantly lower risk of spontaneous preterm birth, while Filipinas had elevated risk

^a^ACOG: American College of Obstetricians and Gynecologists.

^b^PM_2.5_: particulate matter 2.5.

### Limitations

A knowledge of the strengths and limitations of this study is crucial for the effective and appropriate use of these data. With respect to limitations, the lower proportions of linkages to infant hospital records among gestations of <37 weeks reported in our study was similar to the findings from prior population-based linkage studies [31,50]. Greater success of linkage to the records of the birthing person compared to the infant records in these early-gestation pregnancies may be driven by a higher incidence of fetal death in this group. In fact, a prior study recommends excluding births of <24 weeks of gestation from analyses due to the potential misclassification of stillbirths as live births [31]. In our analyses using the SOMI data, we exclude births with gestational age of <22 weeks in all our analyses. Second, Harron [20] defines 2 parameters for linkage accuracy: missed links (2 individual records link, but they are not linked) and false links (2 records from different individuals are linked). We were not able to estimate these linkage accuracy parameters in our study because we do not have access to the electronic medical records informing the HCAI and California Vital Statistics data, and we have no other data source to confirm the correct link. Although we strove to gain better ascertainment of variables such as diabetes and hypertension by linking California Vital Statistics to HCAI records, we are unable to validate the linkage; therefore, true ascertainment is unknown. However, we can state that the rates of these variables increase with the linkage. Third, as previously noted, if twins or multiples shared the same sex, birthweight range, and mode of delivery, and none died or all died, it was impossible to decipher which record should be linked with which infant. This is an unavoidable limitation of this linkage, which must be considered when conducting analyses that include twins or multiples.

The data platform is missing variables that are important for some investigations, including medication use, laboratory values, timing of diagnoses, diagnoses not specified in *ICD* codes, and outpatient visit information. Infants delivered outside of the hospital (eg, home births) could not be linked to a discharge record for the birth, nor could the birthing person.

### The Future of SOMI

SOMI scholars have been recruited to use the data platform to advance clinical knowledge and provide analytic experience for emerging researchers. Scholars have included master’s students, PhD students, medical residents, and fellows. These scholars identify a research question, develop an analytic plan, participate in the data analysis, and present their data at meetings or as a manuscript. Some of these studies have served as thesis projects or part of dissertations [51].

Future projects include using the data platform to obtain additional California state data that will provide information about outpatient visits, medication use, and childhood outcomes beyond 1 year. We also expect that the SOMI data platform will be used to further additional work focused on understanding racial and ethnic as well as socioeconomic disparities in maternal and child health outcomes across San Diego County and California, while also enabling an examination of trends over time by multidisciplinary researchers, policy makers, and other stakeholders.

## Abbreviations

HCAI: California Department of Health Care Access and Information
ICD: International Classification of Diseases
OSHPD: California Office of Statewide Health Planning and Development
SFN: state file number
SOMI: Study of Outcomes in Mothers and Infants
SSN: social security number
UCSD: University of California San Diego
UCSF: University of California San Francisco

## Data Availability

The datasets generated and analyzed during this study are not publicly available due to privacy and confidentiality restrictions because they were collected in accordance with the ethical guidelines of the California Committee for the Protection of Human Subjects for this study but are available for request from the California Department of Health Care Access and Information [21] and California Vital Statistics [12].
